# CD45-targeted pan-leukocyte imaging: mapping whole-body inflammatory activity with specificity and translational promise

**DOI:** 10.1038/s41392-025-02504-4

**Published:** 2025-12-04

**Authors:** Seung Ho Baek, Jung Joo Hong

**Affiliations:** 1 National Primate Research Centre, Korea Research Institute of Bioscience and Biotechnology (KRIBB), Cheongju, Chungcheongbuk Korea; 2 KRIBB School of Bioscience, Korea University of Science & Technology (UST), Daejeon, Korea

**Keywords:** Molecular engineering, Imaging

In a study published recently in *Nature*, Salehi Farid and colleagues introduced CD45-PET, a pan-leukocyte imaging strategy targeting CD45, a highly abundant antigen, on nucleated hematopoietic cells, achieving sensitive, specific, quantitative whole-organ inflammation mapping that correlated with disease severity and outperformed 18F-FDG PET in preclinical models.^1^ With routine-compatible chemistry and workflows, CD45-PET is positioned as a promising candidate for first-line clinical use in diagnosis, monitoring, and therapy guidance.

Inflammation drives morbidity across diverse conditions; however, clinicians lack a robust, non-invasive readout of the cellular processes that define active disease. Current practice relies on symptoms, serum surrogates, or anatomy-centric imaging, which misses molecular pathobiology. Molecular imaging with PET offers whole-body functional maps; however, the available tracers trade sensitivity for specificity. ^18^F-FDG PET is widely used and sensitive; however, its uptake by any glucose-avid tissue yields false positives, complex preparation protocols (fasting and dietary manipulations), and limited disease stratification. Conversely, highly specific probes that bind to single pathways or cell subsets often miss relevant inflammation outside their narrow scope or fail when the biology shifts during treatment.^2–4^

CD45 is a tyrosine phosphatase expressed on all nucleated leukocytes across the lymphoid and myeloid compartments. Based on its high copy number, consistent expression in inflamed tissues, and non-internalizing behavior upon antibody engagement, CD45 was tested as a target to unify inflammation. Small antibody fragments labeled with Zirconium-89 (^89^Zr) were engineered to create mouse and human CD45-PET probes optimized for rapid tissue penetration, high affinity, and next-day imaging, and designed to recognize all CD45 isoforms. Engineering details were species-specific: the mouse probe (VHH nanobody) employed site-specific sortase conjugation of deferoxamine (DFO) followed by PEGylation, which preserved affinity and reduced renal background; the human probe (BC8-derived minibody, ~80 kDa) was produced in HEK293 cells and DFO-labeled for ^89^Zr without PEGylation or sortase steps. The 3.3-day half-life of ^89^Zr enables imaging at a delayed time point, improving the target-to-background ratio in tissues.

**Fig. 1:**
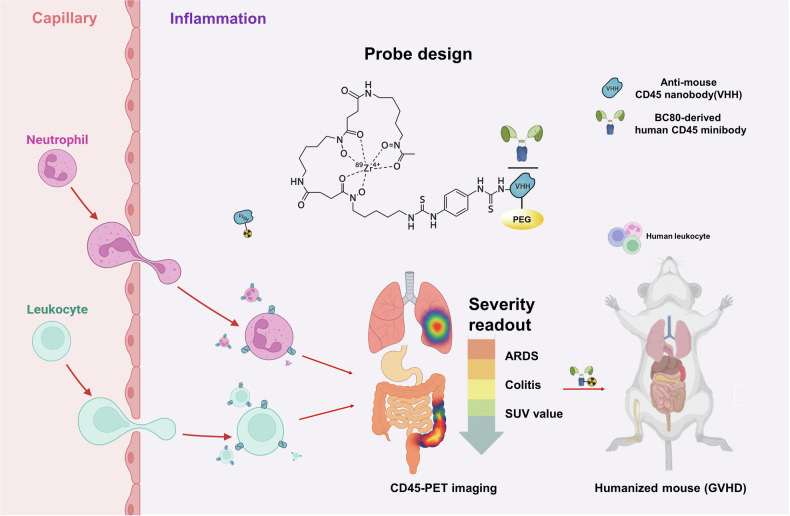
Concept and use‑cases for CD45‑PET. A small-fragment, ^89^Zr-labeled anti-CD45 probe implemented as a mouse VHH nanobody and a BC8-derived human minibody with site-specific DFO conjugation and optional PEG to reduce renal background binds the high-copy, non-internalizing pan-leukocyte antigen CD45 on extravasated leukocytes to generate a quantitative PET signal that maps organ-level inflammatory burden (lung, bowel) and provides lesion SUV value based severity readouts that track histopathology and clinical indices in models of acute respiratory distress syndrome (ARDS) and inflammatory bowel disease (IBD); in humanized mice (graft-versus-host disease, GVHD), the human probe detects engrafted human leukocytes, supporting clinical translation. The figure was created using Biorender.com

Specificity was established using blocking experiments and nontargeting but structurally similar probes. In healthy mice, CD45-PET generated an atlas of immunecell-rich organs and marrow that matched known physiology. Importantly, increased vascular permeability, which is common in inflamed tissues, did not drive nonspecific uptake under the optimized imaging interval, supporting accurate target-mediated signals rather than hemodynamic artifacts. These controls strengthen the claim that the probe binds to CD45 in vivo and that the measured signal reflects the leukocyte burden (Fig. 1).

Across two disease models, acute respiratory distress syndrome (ARDS) and inflammatory bowel disease (IBD) colitis, CD45-PET signal tracked histopathology and clinical indices, and enabled longitudinal response assessment during treatment. In the colitis model, decreases in the large bowel signal mirrored clinical improvements and reductions in disease activity scores, whereas the rectal signal showed weaker concordance, highlighting that organ-level metrics may differ within a compartment. Together, these data indicate that CD45-PET can quantify the inflammatory burden and capture the trajectory, rather than offer a binary ‘hot vs. not’ assessment, which is essential for therapy monitoring.

Head-to-head comparisons with ^18^F-FDG PET underscored the complementary mechanisms and diagnostic advantages of CD45-PET. FDG uptake depends on glucose metabolism, which varies by cell subset and activation state, and physiological uptake can confound interpretation in organs such as the heart and vasculature. Strict dietary preparation can mitigate the background, yet is often impractical for acute illness.^5^ In contrast, CD45-PET integrates signals across the leukocyte compartment without preparation and, in both ARDS and colitis, better stratifies disease severity. In an ARDS model, CD45-PET showed superior performance over a CD11b-targeted probe and uniquely resolved high- versus low-dose LPS groups, whereas CD11b-PET did not.^1^ Mechanistically, the favorable performance is best explained by the high and stable antigen density on leukocytes, although CD45 is largely non-internalizing and preserves surface accessibility during the imaging window, receptor internalization can, under certain tracer-target kinetic conditions, increase cellular trapping and retention.

To enable translation, a human CD45 probe was derived from a BC8 antibody clone that was previously used in clinical radiotherapeutics and cell depletion platforms. In humanized mice, the human CD45-PET probe detected engrafted leukocytes and supported organ-level classification rules based on pre-specified SUV thresholds, which were verified by immunohistochemistry. These data provide a pragmatic bridge from preclinical efficacy to early human imaging and suggest that quantitative readouts can be standardized for decision-making.

This study has two limitations. First, because CD45 is pan-leukocytic, this approach cannot resolve specific immune cell subsets. For indications that require cell type attribution—for example, CD8+ T cell tracking during cancer immunotherapy—subset-selective probes remain necessary.^3^ Second, lymphoid organs harbor a high baseline signal that can mask incremental inflammation, and as with PET, small foci are subject to partial volume underestimation. These caveats define the clinical niches in which CD45-PET will be the most informative: non-lymphoid organs and diseases with an organ-level burden and trajectory to follow.

The cases for clinical use of this approach are clear. CD45-PET could support safer diagnosis in conditions where biopsy is risky or sampling error is common, guide targeted biopsy to high-yield regions, and deliver whole-organ assessments for grading syndromes such as graft-versus-host disease. As a quantitative endpoint, it can accelerate trials of anti-inflammatory agents by providing early pharmacodynamic readouts. Because the original study reports no preclinical dosimetry, translation should proceed with a staged dosimetry program—animal organ/marrow kinetics, model-based human dose estimates, and first-in-human dosimetry. In parallel, the next steps include test–retest reproducibility, reader studies against the current standards of care and multicenter harmonization of acquisition and analysis pipelines to ensure portability across scanners and sites.

Collectively, this study reframes inflammation imaging around the leukocyte compartment rather than any single pathway. A pan-immune target with clinic-ready chemistry and next-day imaging makes CD45-PET a strong candidate for first-line assessment, with the potential to standardize the detection, staging, and monitoring of inflammatory diseases in practice while opening a path to hybrid strategies that overlay CD45-PET with selective tracers for mechanistic depth.
